# Investigation of Compressive and Tensile Behavior of Stainless Steel/Dissolvable Aluminum Bimetallic Composites by Finite Element Modeling and Digital Image Correlation

**DOI:** 10.3390/ma14133654

**Published:** 2021-06-30

**Authors:** Xiuhui Li, Morteza Ghasri-Khouzani, Abdoul-Aziz Bogno, Jing Liu, Hani Henein, Zengtao Chen, Ahmed Jawad Qureshi

**Affiliations:** 1 Department of Mechanical Engineering, University of Alberta, Edmonton, AB T6G 1H9, Canada; xiuhui1@ualberta.ca (X.L.); ghasrikh@ualberta.ca (M.G.-K.); 2 Department of Chemical and Materials Engineering, University of Alberta, Edmonton, AB T6G 1H9, Canada; bogno@ualberta.ca (A.-A.B.); jing22@ualberta.ca (J.L.); hhenein@ualberta.ca (H.H.)

**Keywords:** selective laser melting (SLM), lattice structure, bimetallic composite, mechanical properties, finite element analysis (FEA), digital image correlation (DIC), hybrid manufacturing

## Abstract

This study reports fabrication, mechanical characterization, and finite element modeling of a novel lattice structure based bimetallic composite comprising 316L stainless steel and a functional dissolvable aluminum alloy. A net-shaped 316L stainless steel lattice structure composed of diamond unit cells was fabricated by selective laser melting (SLM). The cavities in the lattice structure were then filled through vacuum-assisted melt infiltration to form the bimetallic composite. The bulk aluminum sample was also cast using the same casting parameters for comparison. The compressive and tensile behavior of 316L stainless steel lattice, bulk dissolvable aluminum, and 316L stainless steel/dissolvable aluminum bimetallic composite is studied. Comparison between experimental, finite element analysis (FEA), and digital image correlation (DIC) results are also investigated in this study. There is no notable difference in the tensile behavior of the lattice and bimetallic composite because of the weak bonding in the interface between the two constituents of the bimetallic composite, limiting load transfer from the 316L stainless steel lattice to the dissolvable aluminum matrix. However, the aluminum matrix is vital in the compressive behavior of the bimetallic composite. The dissolvable aluminum showed higher Young’s modulus, yield stress, and ultimate stress than the lattice and composite in both tension and compression tests, but much less elongation. Moreover, FEA and DIC have been demonstrated to be effective and efficient methods to simulate, analyze, and verify the experimental results through juxtaposing curves on the plots and comparing strains of critical points by checking contour plots.

## 1. Introduction

Recently, lattice structures have attracted the attention of many researchers due to properties such as light weight, high strength, energy absorption, reduced material consumption, and biocompatibility. Lattice structures are formed mathematically or geometrically by spatial arrangement and combination of a grouping of unit cells. Most researchers focus on the mechanical properties, such as compression and tension behavior [1,2,3,4,5,6,7,8], fracture behavior [9,10], fatigue behavior [11,12], and shear response [13], and biocompatibility [14,15,16] of these cells. Research has also been dedicated to design methods of lattice structures, including creating functionally graded porous structures [4,17,18,19], panel or sandwich-shaped lattice structures [20,21,22], and the mathematically designing algorithm [23,24,25,26,27,28].

**Figure 1:**
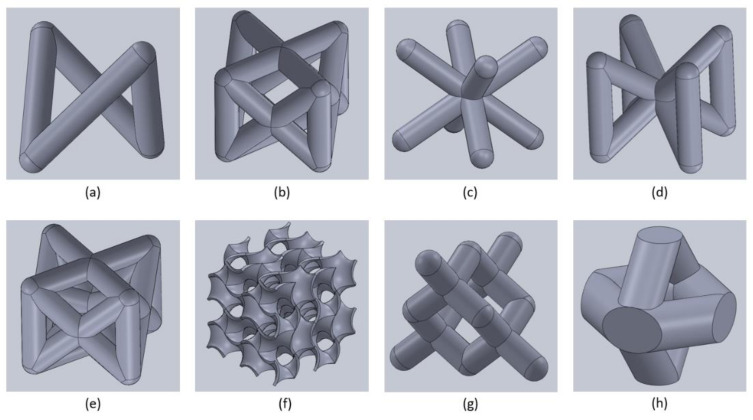
Unit cells in lattice structures: (**a**) FCC; (**b**) F2CC,Z; (**c**) BCC; (**d**) BCC,Z; (**e**) F2BCC,Z; (**f**) gyroid; (**g**) CD; (**h**) Ansys Space-Claim™ diamond.

Most work done on lattice structures has been about unit cells. Researchers were more likely to conduct experiments on normal unit cells formed by the spatial arrangements of struts. However, some of them shed light on complicated unit cells, whose composition components conform to specific mathematical algorithms, such as gyroid [1,3,5,18,19], Schwarz diamond [17,29] called TPMS (triply periodic minimal surfaces), and plate lattices [25]. Compression and tension tests were applied in studying F2CC,Z (face-centered cubic with Z-struts), hollow spherical unit cells by Kohnen et al. [30], and concluded that the mechanical properties for F2CC,Z are better than hollow spherical. Contuzzi et al. [31] studied F2CC,Z structure, and compressive testing using two samples of different volume fractions and concluded that increasing strut thickness is more significant than introducing reinforcement in the lattice structure. Rehme et al. [32] investigated not only F2CC,Z, but also FCC (face-centered cubic) and F2BCC,Z (body-centered and face-centered cubic combined with Z-struts) structures. The difference between these three face-centered cubic unit cells can be seen in Figure 1a,b,e. BCC (body-centered cubic), BCC,Z (body-centered cubic with Z-struts), gyroid and rhombic were also analyzed [2,3,12,29,33,34,35] through compressive, tensile, and fracture testing. They concluded that F2CC,Z has a higher load capacity, and gyroid can be very useful in applications requiring high stiffness. Peto et al. [36] and Park et al. [4] also gave an eye on other kinds of unit cells, which are relatively uncommon and not widely applied, and finally found that CD (cubic diamond) exhibited higher strength compared to others. An image of some unit cells mentioned above is shown in Figure 1. All of these are self-supported for 3D printing except FCC and CD.

Among all unit cells, diamond unit cells are considered the best choice for structures with strength requirements. With predictions of the Gibson-Ashby model, research done by Maconachie et al. [29] evidenced that diamond lattice structures exhibit larger relative strength and relative modulus in the same volume fraction of lattice. However, traditional diamond unit cells, namely CD unit cells, are not self-supported, which might cause some problems in fabrication through additive manufacturing; hence, another type of diamond unit cell inspired by ANSYS Space-Claim™ is plotted in Figure 1h. This diamond unit cell was shown in the lattice auto-generating feature in the Space-Claim, yet there is limited research literature on its properties. Consequently, this diamond unit cell was chosen for the lattice structure in our study.

The manufacturing method of lattice structures has also received widespread attention with metal additive manufacturing (MAM) being a feasible option given the complexity of the geometry. MAM can directly print a geometry layer by layer on a substrate from the bottom to up by metal material feedstock. The sample can be printed from a computer-aided design (CAD), although there are some limitations of samples to be printed in terms of size and geometry for different machines. Selective laser melting (SLM) is one of the categories of MAM. In SLM, thin layers of atomized fine metal powder are evenly distributed using a coating mechanism onto a substrate plate. Then, each layer of the part geometry is fused by selectively melting the powder, which is achieved with a high-power laser beam. Some researchers investigate the defects of the structures fabricated by SLM or AM (additive manufacturing). It was noted that struts waviness, strut oversizing or strut thickness variation is prevalent on lattice structures fabricated by SLM [9,37,38,39,40,41] with horizontal struts features showing more severe geometric imperfections than vertical struts and diagonal struts [9,39,41,42]. Moreover, vertical struts were found to be thinner than as-designed ones [9,37,39], and the magnitude of strut oversizing can change the failure mode from one to another [9]. SLM parameters also affect the mechanical properties of lattice structures [3,43]. Horizontal struts are the first to fracture, indicating they are experiencing greater stress than neighboring struts [41,44].

Although there are some flaws in the structure fabricated by SLM, evidence shows that SLM lattice structures manufactured from stainless steel powder have excellent mechanical performance [32]. Microstructural and mechanical characterizations of duplex stainless steel UNS S31803 processed by SLM was conducted by Hengsbach et al. [45], who validated the successful fabrication of duplex stainless steel processed via SLM. In addition to duplex stainless steel, 316L stainless steel also has been favored by researchers. The mechanical properties and deformation behavior of 316L stainless steel lattice structures fabricated by SLM were studied [30,46], as well as fracture toughness [3]. Bimetallic lattice composites have slowly been gaining interest with researchers. This latticed composite contains two parts, namely the lattice and the matrix, in which another material is filled into the lattice gaps. There is also much research on the microstructure and mechanical properties of bimetallic lattice structures manufactured by SLM, such as CuSn/18Ni300 bimetallic porous structures [47], and A356/316L interpenetrating phase composites [48,49], in which [49] investigated the mechanical properties of PrintCast composites through finite element analysis (FEA), coupled with digital image correlation (DIC) to capture the deformation and failure processes.

FEA is commonly used for simulating the experimental process and validating testing results. Researchers usually conducted FEA for performance evaluation [50,51,52], structure design [53], investigating configurational effects [54], and studying the failure mechanism [55,56]. Digital image correlation (DIC) is a 3D, full-field, non-contact optical technique to measure contour, deformation, vibration, and strain on almost any material. DIC setting is essential for investigating strain rate by analyzing captured images, and it is also apparent to show elongation changing along with the experiments processing. Limited research was done for analyzing deformation and strain evolution applying DIC on stainless steel such as 316L [30,49,57]. Mostly, the focus has been on studying titanium alloy Ti6Al4V [58,59,60,61]. Other investigations into displacement, velocities, and stress measurements using DIC were also done on polymers [62], glass fibers [63], and other materials [64,65].

In this study, FEA and DIC are used to investigate the mechanical properties of 316L stainless steel/dissolvable aluminum bimetallic composites, which are vital for simulating and recording experimental processes. 316L stainless steel lattice structures formed by the unit cell shown in Figure 1h were built using the SLM method, and a molten aluminum alloy infiltrated the 316L stainless steel lattice gaps to create the bimetallic composite. Mechanical properties were analyzed thoroughly by both tension and compression tests, and the experimental results were compared with those from FEA to validate its effectiveness. Simultaneously, the DIC system was also applied to capture strain distribution and verify the FEA results. The following section provides the details of materials and methods used. Section Three describes the FEA simulation model and experimental validation for individual lattices and filler structure. This is followed by Section Four, which details the FEA simulation and experimental validation of bimetallic composite strcutures. Finally, Section Five provides conclusions.

## 2. Materials and Methods

### 2.1. Manufacturing

To study the mechanical properties of materials and structures, both compression and tension tests were performed. Hence, bulk samples, lattice samples and bimetallic composite samples were required for both tension and compression tests. Stainless steel 316L, lattice samples were printed through an EOS M290 machine (EOS, Krailling, Germany), while a proprietary aluminum alloy supplied by the industrial partner was used for the filled-in matrix part of composite by casting. Bulk aluminum samples were also fabricated by casting.

**Figure 2:**
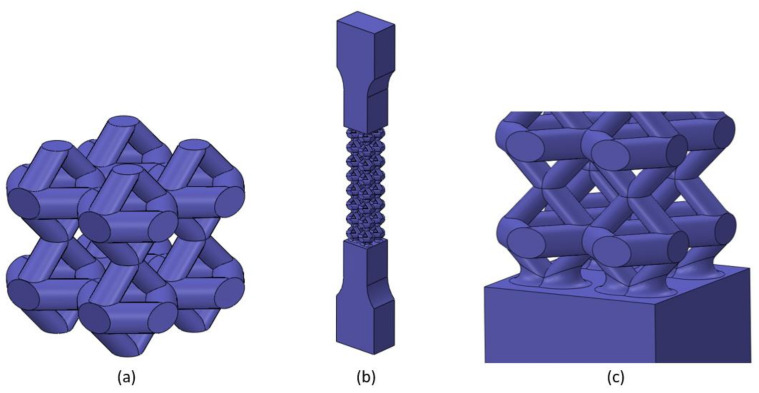
Computer-aided design models (CAD) of the Space-Claim diamond lattice structure parts: (**a**) compression model; (**b**) tension dog-bone model; (**c**) fillets in the interface of dog-bone model.

Compression samples of lattice were in the shape of a cube with a length of 12.5 mm. The tension samples of lattice were a dog-bone shape, whose dimensions conformed to ASTM E8M standard [66], with a gauge length of 50 mm and a gauge width of 12.5 mm. The lattice structure unit cell’s strut diameter is 2 mm, which is the same for both the compression and tension samples. Failure of the tension samples should occur in the gauge zone rather than the interface between the diamond lattice part and the solid gripping part, which is the location of stress concentration. Therefore, fillets were designed on the junction interface of grips to reduce the concentrated stress and avoid failure in this area. The 0.75 mm fillets of the tension sample and the compression sample are displayed in Figure 2. The chemical composition of gas atomized 316L stainless steel powder for the SLM process is listed in Table 1. Tension lattice dog-bone samples were fabricated in a horizontal orientation to the building plate (a hot-rolled mild steel panel with a dimension of 252 mm × 252 mm × 25 mm). EOS Company recommended processing parameters were applied for the 316L stainless steel, and the detailed parameters are listed in [67].

Bimetallic composite samples were manufactured based on the lattice ones. For both compression and tension composite samples, dissolvable aluminum alloy was filled into the lattice structure gaps and formed a matrix part of the composite by the casting process. The chemical composition of dissolvable aluminum is shown in Table 2, and the details for the casting process are presented in Section 2.2 of [67]. Bulk aluminum samples were also fabricated under the same casting condition.

**Figure 3:**
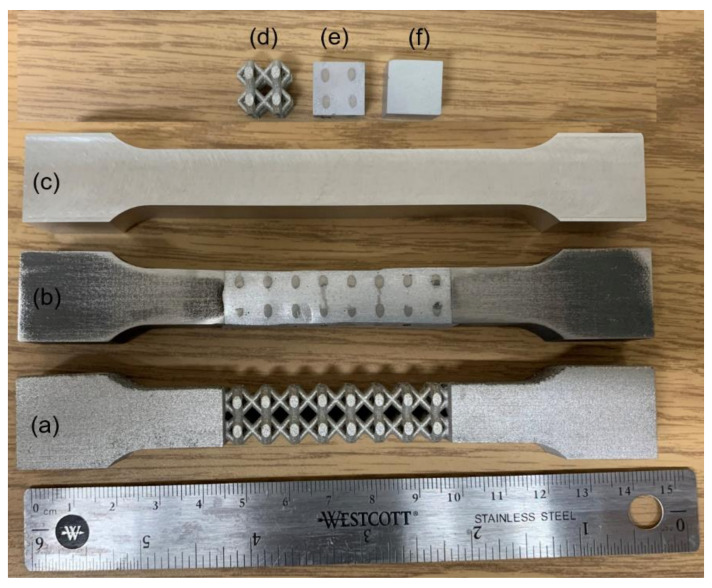
An image of the experimental samples: (**a**) stainless steel lattice dog-bone; (**b**) stainless steel/aluminum composite dog-bone; (**c**) bulk aluminum dog-bone; (**d**) stainless steel lattice cube; (**e**) stainless steel/aluminum composite cube; (**f**) bulk aluminum cube.

Microstructure analysis for the specimens can be found in Section 2.3 of [67]. An image of all the experimental samples is presented in Figure 3.

### 2.2. DIC System Setting

In our experiments, VIC-Snap commercial software (V8, manufactured by Correlated Solutions, Inc., Irmo, SC, USA) was used to capture images, and VIC-3D commercial software (V8, manufactured by Correlated Solutions, Inc., Irmo, SC, USA) was applied to process the images.

Two Allied Vision Technology (AVT) Pike F421b cameras (resolution of 2048 (H) × 2048 (V), sensor size: type 1.2, (Allied Vision Technologies GmbH, Stadtroda, Germany), equipped with two Nikon 28–85 mm F-mount lenses by two C to F-mount adapters (for lenses, Nikon, Tokyo, Japan), which allow for the adjusting of aperture, focus, and zoom, were mounted on a tripod and used in the experiments. Both two lenses provide an average magnification of 10 pixel/mm. One of the cameras was precisely positioned with its lens perpendicular to the focused surface of the lattice sample during the experiments. The other camera’s lens was positioned at 25° to the primary camera. The testing images were captured at the rate of one frame per second, with each frame capturing a compression displacement at around 8 μm and a tension displacement around 33 μm according to the loading speed of 0.5 and 2 mm/min, respectively. The specimens were sprayed with black and white paint (Rust-Oleum, Evanston, IL, USA) to form a scattered speckle pattern on the focused surface with an average diameter of speckles of about 1.3 mm (approximately 5 pixels). Before capturing testing images, a calibration target card with 8 × 8 dots was imaged simultaneously by rotating to different angles in both cameras to calibrate the system in one step thoroughly.

### 2.3. Mechanical Testing

Uniaxial compression and tension tests at room temperature were conducted on all the experimental specimens. The displacement-controlling mode was applied on all the tests using a servo-hydraulic mechanical testing system (MTS 810, MTS, Eden Prairie, MN, USA). The cross-head speed was 0.5 mm/min for compression tests and 2 mm/min for tension tests, leading to an initial strain rate of 6.673 × 10^−4^ s^−1^ for both compression and tension experiments. For more details of the mechanical testing, please refer to Section 2.4 of [67].

## 3. FEA Simulation and Experimental Validation of Individual Lattice, and Bulk Structures

### 3.1. FEA Procedure

The FE analysis was conducted using the commercial FE code ABAQUS™/Explicit (2019 version, Dassault Systemes, Vélizy-Villacoublay, France) [68], with simulation models generated using SolidWorks (V2020, Dassault Systemes, Vélizy-Villacoublay, France) [69]. Comparing to ABAQUS™/Standard, ABAQUS™/Explicit solver can better solve the convergent problems for models with complex configurations, especially for lattice structures. Furthermore, it can also readily analyze problems with complicated contact interaction between the independent bodies [49] for the bimetallic lattice structures clarified in Section 4.

The simulation model needs to be imported into ABAQUS™ before conducting the FE analysis. Then, the material parameters such as Young’s modulus, Poisson’s ratio for elasticity, and "true stress" vs. "plastic strain" values for plasticity in the ABAQUS™ property-material module are set up. The plasticity "true stress" vs. "plastic strain" pairs of values for 316L stainless steel were obtained from [70], while data for aluminum alloy were obtained from the bulk aluminum experiments. After setting up the materials, assigning the specific material to the model configuration accordingly, for example, 316L stainless steel, was given to the lattices while aluminum was given to the bulk aluminum models.

For compression model boundary conditions, the bottom end (one surface for bulk models, four small surfaces for lattice models) was fixed for all the six degrees of freedom (U1 = U2 = U3 = UR1 = UR2 = UR3 = 0). Simultaneously, a reference point was generated on the top and coupled with the top end (one surface for bulk models, four small surfaces for lattice models), with five degrees of freedom fixed (U1 = U3 = UR1 = UR2 = UR3 = 0) and one remained (U2) for the loading. A velocity of 0.5 mm/min was then applied to the top reference point in the U2 direction. Note that the applying velocity should not be consistent from the beginning of the analysis until the end. Based on the actual experiment, the loading speed shall change gradually from 0 mm/min initially, to the maximum in the middle, then drop back to 0 mm/min in the end, at which time the average rate would be 0.5 mm/min. In this case, the amplitude of velocity gradually changed throughout the whole loading process. As for tension models, similarly, the bottom end of the dog-bone gripping area was fixed for all degrees of freedom (U1 = U2 = U3 = UR1 = UR2 = UR3 = 0), while a velocity of 2 mm/min was applied to the reference point on the top in the U2 direction (U1 = U3 = UR1 = UR2 = UR3 = 0).

The last step before running the FE analysis was meshing. The free linear tetrahedral 3D stress element (C3D4 element type) was selected for both compression and tension lattice models and tension bulk dog bones, while the structured linear hexahedral 3D stress element (C3D8 element type) without reduced integration was used for compression bulk samples. Note that C3D4 was also used on the gripping block areas of tension lattice models to assure consistency with the lattice part. The mesh size for compression lattice samples is 0.5 mm, and 1 mm for all other models. For the compression bulk 316L stainless steel model, the compression bulk aluminum alloy model, and the 316L stainless steel lattice model, the number of elements are 2197, 2197 and 47,336, respectively, with node numbers of 2744, 2744, and 10,895. For the tension bulk 316L stainless steel model, tension bulk aluminum model, and tension 316L stainless steel lattice model, the numbers of elements are 158,001, 158,001, and 188,681, respectively, with nodes numbers of 30,622, 30,622, and 40,588.

**Figure 4:**
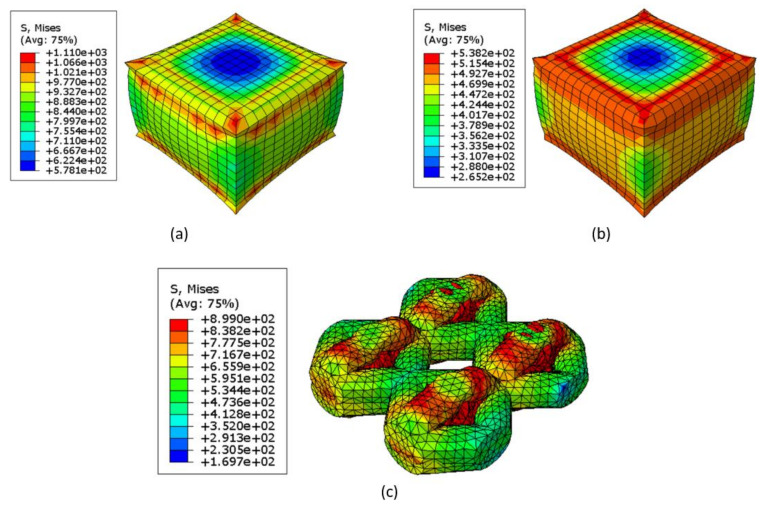
Deformation contour plots of FEA for compression samples: (**a**) bulk 316L stainless steel cube; (**b**) bulk dissolvable aluminum cube; (**c**) 316L stainless steel lattice.

**Figure 5:**
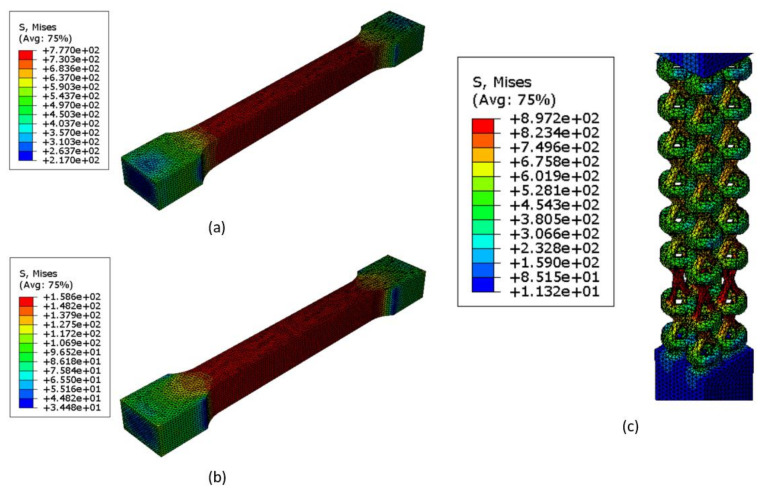
Deformation contour plots of FEA for tension samples: (**a**) bulk 316L stainless steel dog-bone; (**b**) bulk dissolvable aluminum dog-bone; (**c**) 316L stainless steel lattice dog-bone.

Figure 4 and Figure 5 show deformation contour plots for bulk 316L stainless steel, bulk aluminum, and 316L stainless steel lattice under both compressive and tensile conditions. Stresses shown in the plots were all von Mises stress averaging at 75%. The value 75% here means that if the relative difference between contributions that a specific node gets from its neighboring elements is less than 75%, these contributing values are averaged [68]. The local effects on Figure 4a,b might come from the contact boundary condition applied. The rigid plate is used to apply the compressive load to the sample. When the deformation reaches the highest level in compression, friction between the rigid surface and the sample surface will lead to "sticking condition" which leads to much higher result as seen in the model results. This however only accounts for a very limited range of the whole load carrying area. As a result, the actual stress used to represent the bulk behavior of the compression sample is much less than the 1110 MPa as shown. The same situation applies to the Figure 4b. It is also evident that 316L stainless steel is much stronger and can afford more stress than aluminum under both compressive and tensile conditions. Moreover, compressive strength is almost the same as tensile strength for the lattice sample since there is no significant difference between their ultimate stress in the deformed contour plots.

After getting the contour plot, the reaction force and displacement of the top reference point of each model were exported from ABAQUS™ to an excel sheet. The engineering stress ($\sigma_{E}$) and engineering strain ($\varepsilon_{E}$) were obtained using the equations below:

(1) $$\sigma_{E}=\frac{The\;reaction\;force\;\left(N\right)}{The\;failure\;cross\;section\;area\;\left(mm^{2}\right)},\;\left(MPa\right)$$

(2) $$\varepsilon_{E}=\frac{The\;displacement\;\left(mm\right)}{The\;sample\;\left(gauge\right)\;length}$$

**Figure 6:**
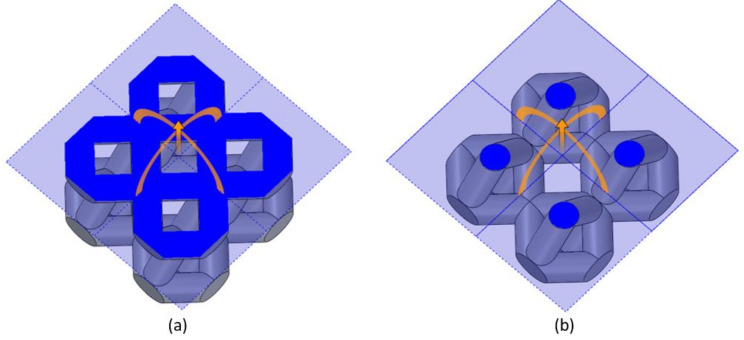
Maximum and minimum areas of the compression lattice model: (**a**) maximum area and (**b**) minimum area.

The compression model is a cube of 12.5 mm in each direction, and the gauge length for all tension models is 50 mm. The cross-section area for both compression and tension bulk models is 156.25 mm^2^ (12.5 mm × 12.5 mm). However, as the cross-section area varies throughout the whole length of lattice samples, the average cross-section area size of 60.99 mm^2^ is adopted with a maximum of 109.42 mm^2^ and a minimum of 12.56 mm^2^. Figure 6 shows the positions of maximum and minimum areas of the lattice using the compression one as the example.

Using the formulas below, we can convert the engineering stress ($\sigma_{E}$) and engineering strain ($\varepsilon_{E}$) to true stress ($\sigma_{T}$) and true strain ($\varepsilon_{T}$):

(3) $$\varepsilon_{T}=\ln\left(1+\varepsilon_{E}\right)$$

(4) $$\sigma_{T}=\sigma_{E}\left(1+\varepsilon_{E}\right)$$

**Figure 7:**
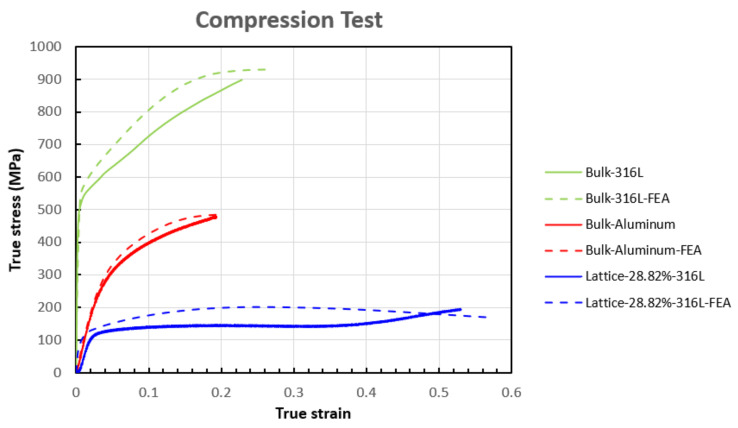
Comparison between experimental and FEA results of bulk 316L stainless steel, bulk dissolvable aluminum, and 316L stainless steel lattice for the compression test.

**Figure 8:**
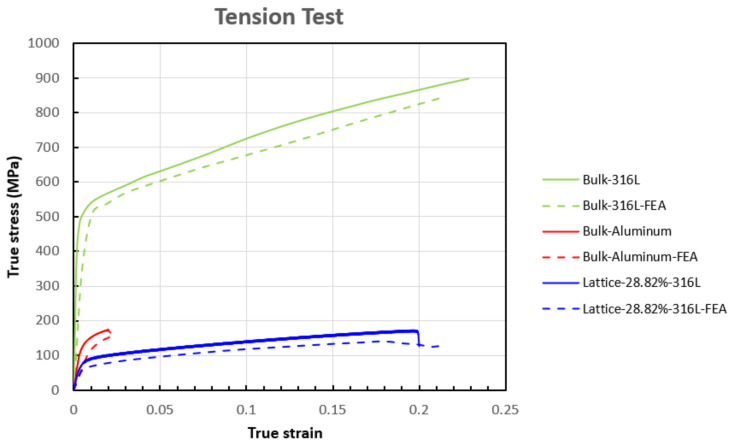
Comparison between experimental and FEA results of bulk 316L stainless steel, bulk dissolvable aluminum, and 316L stainless steel lattice for the tension test.

The “true stress” vs. “true strain” plots for FE compression and tension tests are shown in Figure 7 and Figure 8. The experimental work will be discussed in Section 3.2, and the comparison will be made between the FEA and experimental results to verify the consistency.

### 3.2. Experimental Validation of FEA Results

The experimental 316L stainless steel data was obtained from [70]. Overlapping the FEA compression plot in Section 4.1 to this experimental plot, we then obtained the final comparison plot between the FEA result and experimental result for all bulk and lattice specimens shown in Figure 7. We can see that for the three materials, the FEA results and experimental results are in conformance with each other, with average calculated numerical deviations of 9.8 and 5.0% for yield stress and ultimate compressive stress, respectively. Although the general shape of the Lattice-28.82%-316L with Lattice-28.82%-316L-FEA curves are in agreement, in certain areas, the curves show a difference. This difference becomes more apparent as the plastic deformation increases. These differences occur due to unavoidable manufacturing and material defects, such as microporosity, surface roughness, deviation from the nominal dimensions, and the offset of the strut axes from the ideal axes. These variations will affect the mechanical strength of samples. Macrostructure based finite element model as presented in this work has not integrated these defects. Therefore, consequently, the FEA results are overestimated compared to the corresponding experimental results. As the specific sample is a lattice structure with a high volumetric void ratio, these errors seem higher. However, as is apparent in Figure 7, as the void volume ratio decreases, these errors also significantly decrease. These errors also decrease as the plastic deformation progresses towards the end where the sample densification occurs. Moreover, it is also obvious that the yield and ultimate compressive stress of 316L stainless steel lattices are less than those of both the bulk aluminum and the bulk 316L stainless steel, which means the strength of the lattice with a volume fraction of 28.82% is significantly less than the solid samples due to low volume fractions. The ultimate compressive stress, which represents the compressive strength of the lattice, can be significantly enhanced by increasing the lattice strut diameter [31]. Furthermore, the cracks in the microstructure of the lattice can also explain the much lower yield stress and compressive strength.

Moreover, Figure 7 shows that the compression test for bulk aluminum stopped much earlier than the 316L stainless steel lattice counterpart. This is due to the test being stopped at the load limit (100 kN) of the mechanical testing machine before the specimen failure, while the 316L stainless steel sample collapsed before the test stopped. Three significant deformation stages, which are the elastic stage, plateau stage and densification stage, are shown in the 316L stainless steel compressive curve compared with the bulk aluminum. Initially, lattice struts were in an elastic deformation stage under the compressive load. Then, the struts approached the yield point, and the plastic stage began, which is indicated as the plateau stage. In the plateau stage, the strut nodes were dramatically squeezed, and plastic hinges formed. Finally, the densification started since the struts were continuously compressed to the point where some were broken, while others were closely squeezed against each other.

Identically, the experimental 316L stainless steel data were also collected from [70]. In order to be consistent with the compression result and further compare with the FEA result, all the experimental engineering values were transformed to the true values by using Equations (3) and (4). Similarly, mapping the FEA tension plot in Section 4.1 to this experimental plot, we then obtained the final tension plot between the FEA result and experimental result for all bulk and lattice specimens shown in Figure 8. This plot also validates that the FEA results agree with the experimental, with average calculated numerical deviations of 2.1 and 8.9% for yield stress and ultimate tensile stress. Likewise, the yield stress and tensile strength of the 316L stainless steel lattice are much lower than the other two bulk models. Increasing the strut diameter to achieve a bigger volume fraction will also improve the tension property.

Unlike the compression testing, which has three deformation stages, the 316L stainless steel lattice just experienced the initial elastic stage and the elongational plastic stage, followed by fracture failure with a sudden drop in stress eventually. Moreover, the tensile behavior of the bulk aluminum exhibits an apparent difference from the other two, with a higher Young’s modulus than the lattice but much less elongation than the other two. This is because aluminum is more brittle and has lower resistance to the tensile loading than 316L stainless steel, making it much easier to fracture with shorter elongation. In contrast, the diamond lattice configuration achieved a much-extended elongation and can be widely used in the energy absorption structure.

### 3.3. Experimental Validation with DIC Results

**Figure 9:**
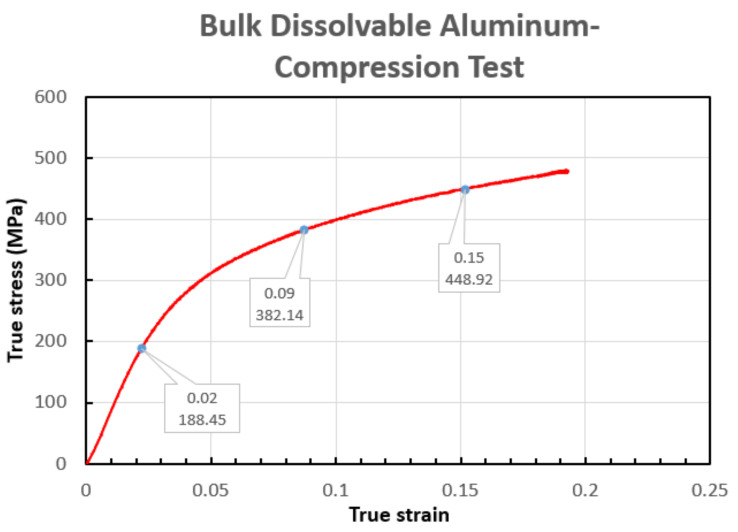
The experimental result of compression bulk dissolvable aluminum cube with three unique points marked out with true stress and true strain.

**Figure 10:**
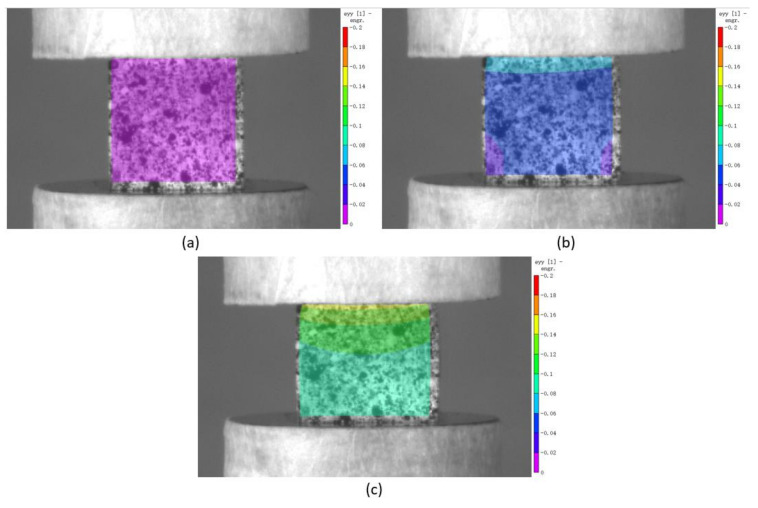
DIC frames of the three points marked out in the bulk dissolvable aluminum compression curve: (**a**) 34 s; (**b**) 131 s; (**c**) 228 s.

As for the comparison between the experimental and DIC results, we discuss the compression bulk aluminum and tension 316L stainless steel dog-bone lattice samples for brevity. A detailed view of bulk aluminum compression experimental curve is shown in Figure 9. Three unique points, namely the yielding point, the point in the plastic region, and the point in the hardening region, were marked out with their true strain and true stress values. The corresponding DIC images to these points are shown in Figure 10.

**Figure 11:**
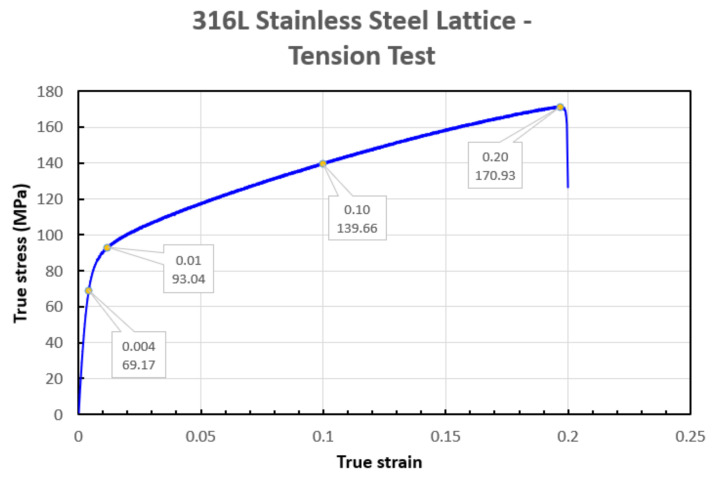
The experimental result of tension 316L stainless steel dog-bone lattice with four unique points marked out with true stress and true strain.

**Figure 12:**
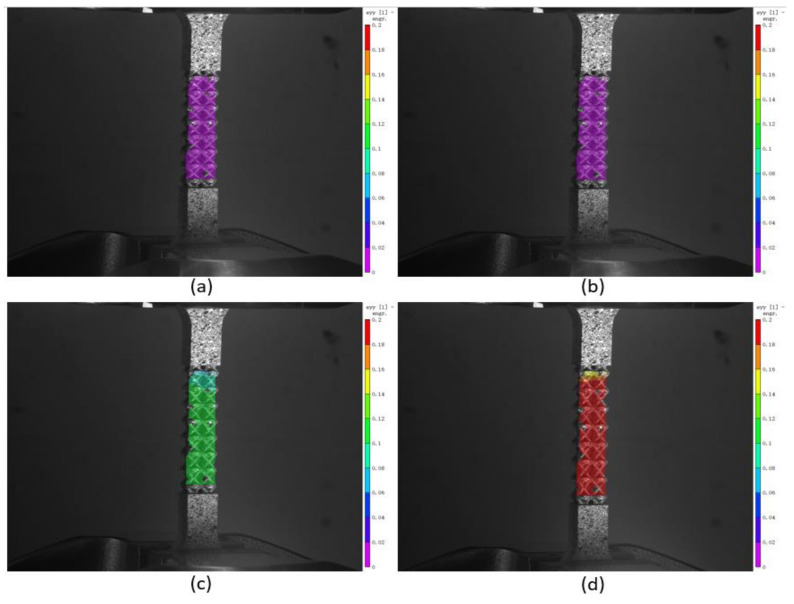
DIC frames of the four points marked out in the tension 316L stainless steel dog-bone lattice curve: (**a**) 6 s; (**b**) 18 s; (**c**) 150 s; (**d**) 295 s.

The scale bar is listed on the right side of each picture, with the strain range of −0.2 to 0 (negative values represent the compression test). From the frames, we can see that the color symbolizing engineering strain changes with loading progression, and the experimental results match the value range as the frames plotted. Figure 10a shows a uniform strain distribution as there is no severe displacement but with the increase in displacement, clear and uneven distribution can be observed in the subsequent Figure 10b,c. Similarly, four particular points, namely the yielding point, the turning point, the point in the plastic region, and the point before the curve drop, are marked out on the tension test experimental curve of the 316L stainless steel dog-bone lattice in Figure 11, with corresponding DIC images shown in Figure 12 in an increasing strain sequence, with strain ranging from 0–0.2. Figure 12a–d show the DIC images corresponding to the four points on the stress strain curve, obtained through the tensile testing machine using an extensometer. DIC shows slight uneven distribution of the strain within the sample gauge length. The highest strain obtained from DIC matches the result from extensometer well. It can also be observed from Figure 12c,d that the strain at the end of the lattice, where it attaches to the solid part of the sample is uneven and much less. This is in accordance with the expectation as the strain decreases with the increasing part density.

## 4. FEA Simulation and Experimental Validation of Bimetallic SS316L-Aluminum Alloy Bimetallic Composite

### 4.1. FEA Procedure

**Figure 13:**
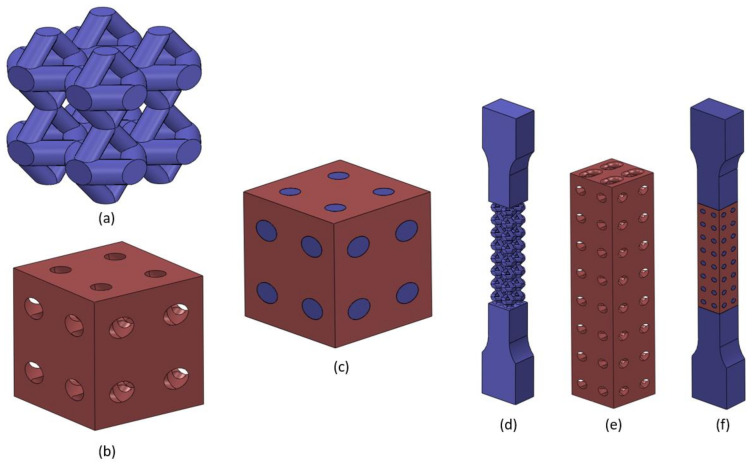
CAD models of the composite parts: (**a**) lattice part for the compression composite; (**b**) matrix part for the compression composite; (**c**) the compression composite; (**d**) lattice part for the tension composite; (**e**) matrix part for the tension composite; (**f**) the tension composite.

For FEA modeling of the bimetallic composite, two separate models were constructed in SolidWorks™ and imported and combined in ABAQUS™. ABAQUS™/Explicit (2019 version) solver was used in this work as it is appropriate to solve problems involving two models contacting each other. Separate models of both compression composite and tension composite created in SolidWorks are shown in Figure 13.

Similar to procedure in Section 3.1, the materials were assigned to the corresponding part of the composite after importing the models into ABAQUS™. Materials for both compression composite and tension composite are the same, namely 316L stainless steel for the lattice part, and aluminum for the filled-in matrix part. Next, separate models were assembled into one composite pattern, and the geometry centers of both the lattice part and the matrix part were ensured to coincide. Setting up interaction between two objects of a composite is critical in ABAQUS™ FEA. Based on the microstructural analysis of the interface as reported in [67], it is observed that there is no cohesive bonding between the two parts, and therefore, a "hard contact" interaction of the 316L/aluminum interface was generated in ABAQUS™. Two surface sets were established, with one set of the outer surfaces of the lattice, and the other of the inner surfaces of the matrix, to be selected for creating the surface interaction. No penetration in the normal direction is assumed, and isotropic friction with a coefficient of 0.3 in the tangential direction is applied without elastic slip and any other shear stress for both the compression and tension composite patterns. Finally, a reference point is created on the top surface and coupled with the top cover for applying the load.

The boundary conditions for both compression and tension composites are the same as the models for bulk and lattice experiments. The bottom end was fixed for all the six degrees of freedom (U1 = U2 = U3 = UR1 = UR2 = UR3 = 0), and the top reference point was held for five degrees of freedom except for U2 (U1 = U3 = UR1 = UR2 = UR3 = 0). A gradually changed velocity of an average of 0.5 mm/min was applied on the reference point for the compression sample, while 2 mm/min for the tension, maintaining consistency with the experiments. Figures of boundary conditions for compression and tension composites are omitted here since there is no significant difference with those shown in Section 3.1.

The free linear tetrahedral 3D stress element (C3D4 element type) was applied to both the lattice and matrix part of compression and tension composites. It is worth noting that the gripping block areas of the tension composite dog-bone also used C3D4, which is identical to the tension lattice dog-bone meshing. The mesh size for the compression composite was 0.5 and 1 mm for the tension composite. Moreover, there are overall 152,845 and 327,547 elements, and 32,891, and 70,978 nodes for the whole compression and tension composites, respectively.

**Figure 14:**
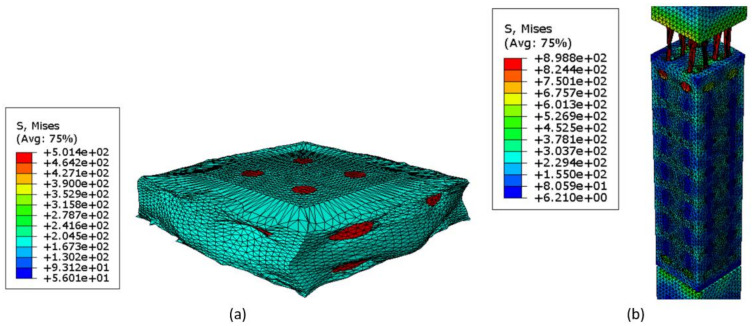
Deformation contour plots of FEA for composite samples: (**a**) compression composite cube and (**b**) tension composite dog-bone.

Figure 14 gives the deformation contour plots of two composites. Stresses shown in the plots were all von Mises stress averaging at 75% of elongation. We can see that the composite is severely deformed under the compressive loading, and the matrix part is in light-green color, which means it afforded the load and played an essential role in resisting the load. In contrast, the tension composite matrix is almost in the blue color. Compared with the scale bar, we know that the insignificant load transferred to the matrix. This is due to a lack of interface fusion due to continuous cracks in the 316L/aluminum interface preventing the load transfer from the lattice to the matrix.

“Engineering stress” and “engineering strain” were then collected from the reaction force and displacement exported from ABAQUS™ using Equations (1) and (2), and corresponding “true stress” and “true strain” were calculated by Equations (3) and (4). The sample length was 12.5 mm for the compression composite, while 50 mm (gauge length) for the tension composite. The cross-section area was 156.25 mm^2^ (12.5 mm × 12.5 mm) for the compression; however, this is not the case for the tension.

**Figure 15:**
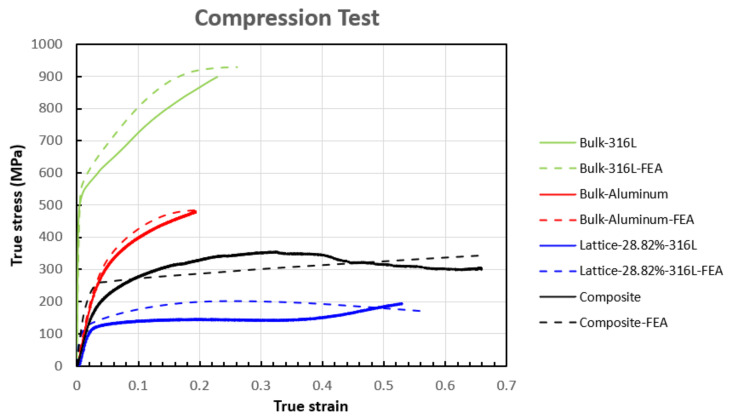
Comparison between experimental and FEA results of bulk 316L stainless steel, bulk dissolvable aluminum 316L stainless steel lattice, and 316L stainless steel/dissolvable aluminum composite for the compression test.

**Figure 16:**
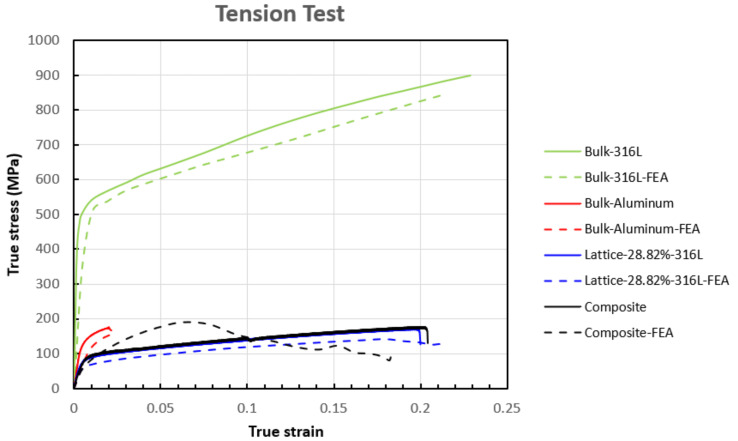
Comparison between experimental and FEA results of bulk 316L stainless steel, bulk dissolvable aluminum 316L stainless steel lattice, and 316L stainless steel/dissolvable aluminum composite for the tension test.

The “true stress” vs. “true strain” plots for compression and tension composite FEA results are shown as dashed black lines in Figure 15 and Figure 16, respectively in Section 4.2 for comparison. Similarly, the experimental work will also be discussed, and the comparison will be made between the FEA and experimental results to verify the consistency.

### 4.2. Experimental Validation of FEA Results

“True stress” vs. “True strain” curves of experimental results of the composite at room temperature as well as FEA results are plotted with other results of bulk and lattice samples in Figure 15 and Figure 16 for compression and tension tests, respectively.

In terms of composite tests, there is a lack of bonding between the aluminum matrix and the SS316 lattice. This lack of material bonding plays a role in the experimental results of the compression as well as tension samples. This interface in the FEA is modeled as a hard contact with a corresponding friction coefficient. This coefficient is a constant value in the model. In the experimental tests, based upon the nature of test, i.e., compression, or tension, the interface between the two materials evolves as a function of strain and loading condition. Based on these differences it can be observed that the FEA results underestimate the compression and overestimate the tension. However, despite these, the calculated numerical deviation of 2.0% for the ultimate compressive stress confirms that the FEA simulation shows a good accuracy. Moreover, it is also apparent from the plot that the yielding and ultimate compressive strength has been significantly enhanced from the lattice shown in blue to the composite shown in black due to the filled-in matrix part. Nonetheless, the mechanical properties of the composite are less than the bulk aluminum properties shown in red. This can be addressed by increasing the volume fraction of the lattice. Using the rule of mixtures, this would result in composite properties between the lower bound of bulk aluminum and the upper bound of bulk 316L stainless steel.

**Figure 17:**
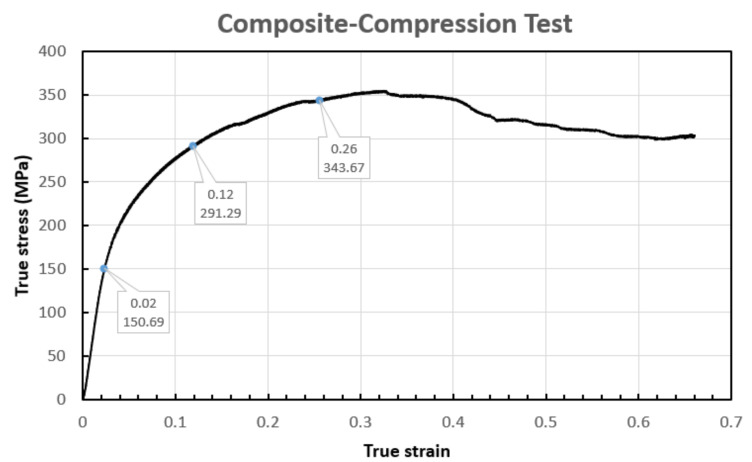
The experimental result of compression composite cube with three unique points marked out with true stress and true strain.

**Figure 18:**
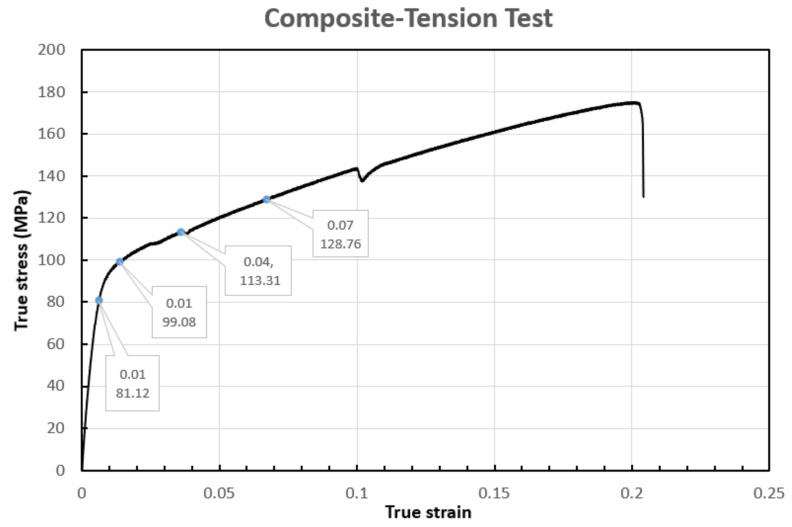
The experimental result of tension composite dog-bone with four unique points marked out with true stre*ss* and true strain.

**Figure 19:**
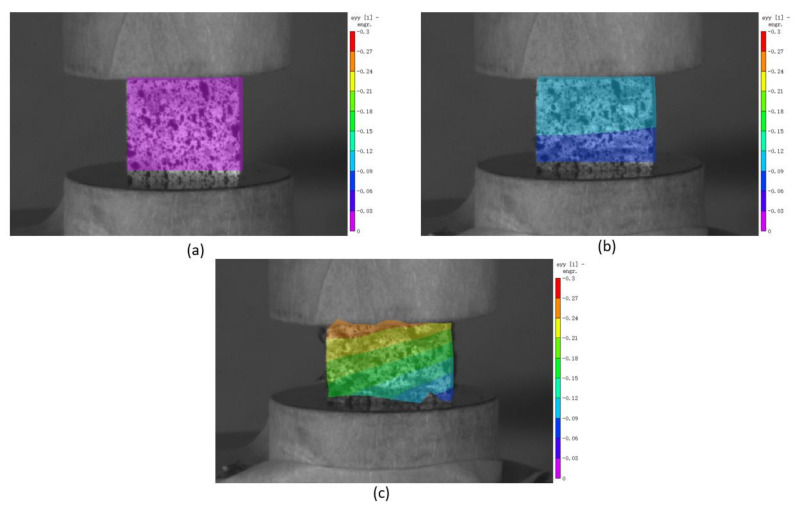
DIC frames of the three points marked out in the composite compression curve: (**a**) 35 s; (**b**) 179 s; (**c**) 383 s.

**Figure 20:**
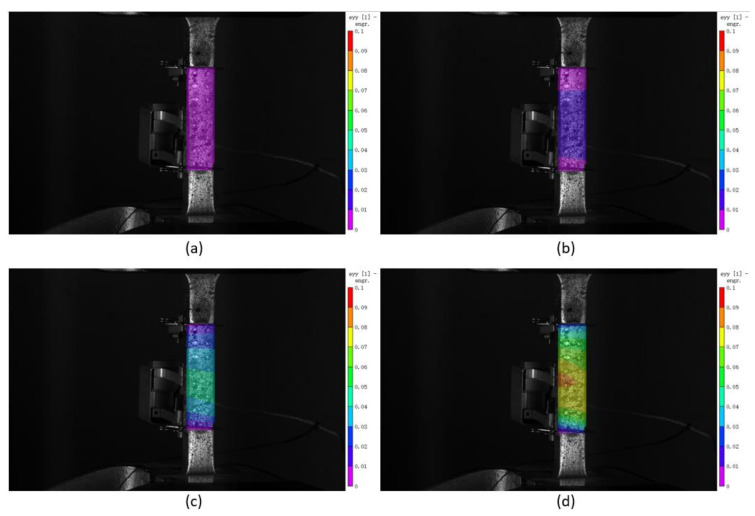
DIC frames of the four points marked out in the composite tension curve: (**a**) 9 s; (**b**) 21 s; (**c**) 54 s; (**d**) 101 s.

Composite compression and tension experimental curves were taken out of the plots shown in Figure 17 and Figure 18. For the compression test, as clarified in Section 3.3, three unique points, namely the yield point, a point in the plastic region, and a point in the hardening region, were marked out with their true strain and true stress values, and the corresponding frames captured by the DIC system are shown in Figure 19. In contrast, for the tensile test, four points, namely the yield point, a point in the plastic region, a point before the first curve dip, and the last point that the DIC effectively tracked, were marked out, and the DIC results were shown in Figure 20. The corresponding time calculated for the compression test was 35, 179, and 383 s, while 9, 21, 54, and 101 s for the tension test.

The tension results are different from the compression curves, where two distinct regions can be found in the experimental results, the elastic region, and plastic region, after which a sudden drop is shown, indicating the rupture of the sample. It is significant to note that the tensile curves for the 316L stainless steel lattice and bimetallic lattice are similar. This indicates that the aluminum matrix does not play an essential role due to lack of bonding. Similar to the compression results, the bulk 316L stainless steel and bulk aluminum possess higher yield stress and ultimate tensile stress, and both tensile curves of the 316L lattice and composite do not even surpass the curve of bulk aluminum. However, the dissolvable aluminum presents a much lower elongation comparing to the other three samples. The trivial difference between the experimental and FEA data for all four pairs validates the simulation results, including the numerical calculated deviation of 2.0% for the ultimate stress of the tension composite. The ABAQUS™ simulation curve for the bimetallic composite generally matches the results from Cheng et al. [49].

### 4.3. Experimental Validation with DIC Results

The DIC data for compression and tension tests of composite samples reveal that the strain pattern is uneven along the length of the sample. This is in departure from the DIC test results for the bulk aluminum, as well as the SS316 lattice structure, which showed a more even strain distribution as compared to the composite samples.

A strain range of −0.3 to 0 (Figure 19) was exhibited in the compression and 0 to 0.1 (Figure 20) in the tension. The strain behavior of the compression composite represented by the color coding was very similar to the bulk dissolvable aluminum. However, slight differences were observed for the tension composite. The strain growth was observed to grow gradually from the center to both sides, initially from 0 shown as purple color in the first frame to about 0.07 with orange color appearing in the middle part of the last frame. Experimental strain results of the curve plots (Figure 17 for compression and Figure 19 for tension) match the value range plotted in the frames for both the compression and tension composite samples.

## 5. Conclusions

The work presented in this study provides a novel and original method to model and simulate bimetallic lattice structures. Bimetallic lattice structures are an emerging field of materials that harness the properties of their constituent materials and provide a meta material capable of engineered functional response. The capability to engineer these meta materials makes them an ideal candidate for applications in biomedical, aerospace, defence, space, and oil and gas industries. The bimetallic composite combination studied and modeled in this specific research work also possesses functional properties due to the dissolvable aluminum alloy matrix, which allows a part of the composite to dissolve while retaining its cellular, lattice-based structure. By investigating the compressive and tensile behaviour of 316L stainless steel lattice, bulk dissolvable aluminum alloy, and 316L stainless steel/dissolvable aluminum bimetallic composite, the following conclusions can be obtained:

1. The developed FEA model is an acceptable simulation for the experimental work. After validating the effectiveness of ABAQUS™ FEA simulation on the current experiments, the simulation can be used to explore different volume fractions of base lattice and filler to obtain desired properties without the need for extensive experiments. For bulk and lattice samples, the average calculated numerical deviations between experimental and FEA results in this study for yield stress and ultimate stress are 9.8 and 5.0% for compressive tests and 2.1 and 8.9% for tensile tests, respectively. For composite samples, the average calculated numerical deviations for ultimate stress are 2.0% for both compressive and tensile experiments. Further improvements to the model can be made by integrating the manufacturing dimensional variations as well as manufacturing induced material imperfections.
2. 316L stainless steel has better compressive properties and higher resistance to the tensile loading than dissolvable aluminum alloy, which is more brittle with less elongation.
3. In the tension test, due to lack of bonding, the load does not transfer from the 316L stainless steel lattice to aluminum alloy. However, the aluminum alloy part plays an indispensable role in the compression test and enhances the composite’s compression strength compared to the lattice itself.
4. The elastic modulus, yield stress, and ultimate stress of both the 316L stainless steel lattice and bimetallic composite were lower than the bulk aluminum, proving that the performance of the lattice and composite with a volume fraction of 28.82% is still not that satisfactory. Increasing the strut diameter of lattice to achieve a higher volume fraction is expected to enhance the mechanical properties, including both compressive and tensile strengths.

## Figures and Tables

**Table 1:** Chemical composition of 316L stainless steel powder used as the feedstock material for the AM process (wt.%).

Chemical Composition	C	Cr	Mn	Mo	N	Ni	O	S	Si	Fe
**Value (wt.%)**	0.03	17.9	2.0	2.4	0.1	13.9	0.04	0.01	0.75	Balance

**Table 2:** The chemical composition of the aluminum alloy used for casting (wt.%).

Chemical Composition	Fe	Ag	Ga	Cu	Mg	Al
**Value (wt.%)**	0.6	2.1	2.0	2.6	4.1	Balance

## Data Availability

Data available on request due to restrictions eg privacy or ethical.
